# The moderate substitution of *Astragalus sinicus* returning for chemical fertilizer improves the N cycle function of key ecological bacterial clusters in soil

**DOI:** 10.3389/fmicb.2022.1067939

**Published:** 2023-01-05

**Authors:** Minghao Lv, Yongdong Wang, Xiaofen Chen, Wenjing Qin, Wencong Shi, Weifeng Song, Jingrui Chen, Changxu Xu

**Affiliations:** ^1^Institute of Soil and Fertilizer and Resources and Environment, Jiangxi Academy of Agricultural Sciences, Nanchang, China; ^2^State Key Laboratory of Crop Biology, Shandong Agricultural University, Tai’an, China

**Keywords:** *Astragalus sinicus*, organic substitution, bacterial community, ecological cluster, nitrogen cycle function

## Abstract

*Astragalus sinicus* (Chinese milk vetch) is a well-established resource of organic fertilizer widely used in paddy soil to partially replace chemical fertilizers. However, the influence of returning *A. sinicus* to fields on the soil bacterial community remains poorly understood. Here, we used different amounts of *A. sinicus* partially replacing chemical fertilizers and investigated the changes in soil physicochemical factors and the soil bacterial community structure responses. Returning *A. sinicus* to the field significantly increased the soil total nitrogen and available phosphorus content (*p* < 0.05). Weighted gene correlation network analysis (WGCNA) was applied to detect significant associations between the soil microbiome data and physicochemical factors. Two key ecological bacterial clusters (MEturquoise and MEgreen), mainly containing Acidobacteria, Proteobacteria, and Chloroflexi, were significantly correlated with soil nitrogen (N) levels. *A. sinicus* partially replacing chemical fertilizers reduced the normalized stochasticity ratio (NST) of rare amplicon sequence variants (ASVs), abundant ASVs, MEturquoise, and MEgreen (*p* < 0.05). Our results further indicated that a moderate amount of *A. sinicus* returned to the soil effectively mitigated the trend of reduced relative abundance of N fixation function of key ecological clusters caused by chemical fertilizer. However, a large amount of *A. sinicus* led to a significant increase in relative abundance of denitrification function and a significant decrease in relative abundance of N fixation function of key ecological clusters. This implies that the moderate substitution of *A. sinicus* returning for chemical fertilizer improves the N cycling function of key ecological bacterial clusters in soil. From the perspective of the bacterial community in paddy soil, this study provides new insight and a reference on how to find a good balance between the amount of *A. sinicus* returned to the soil and ecological safety.

## Introduction

The nitrogen (N) cycle is an important biogeochemical cycle, as N is an essential element for all living organisms and is involved in the biosynthesis of important substances such as nucleic acids and proteins (Fan, 2019; Lehnert et al., 2021). Human activity has a profound impact on global biogeochemical N cycling, mainly because of the large amount of N fertilizer inputs required for food production (Kuypers et al., 2018). Especially in terrestrial ecosystems, N is considered a limiting nutrient (Zheng et al., 2020). Therefore, in many areas, farmers apply too much N fertilizer to the soil to meet the needs of plant growth, while often less than half of this N is absorbed and used by plants, which exacerbates global climate warming and ozone depletion (Lehnert et al., 2021).

Microorganisms are important players in the N cycle. Biological N fixation fixes about 100 Tg of N from the atmosphere annually, whereas the N cycling function, represented by denitrification, has also been considered a key process determining N loss in terrestrial ecosystems (Fang et al., 2015; Fan et al., 2019). Thus, there is an urgent need to investigate the response patterns of soil microorganisms in order to understand the effects of *Astragalus sinicus* returned to the soil. Soil microorganisms are sensitive to environmental changes. Different ecosystems may vary greatly in microbial response to environmental changes because of different management practices (Dai et al., 2018). In turn, the diverse metabolism of microbes may result in various changes in soil environmental conditions. The abuse of chemical fertilizers can lead to the destruction of microbial community structures in farmlands and reduce the abundance of some microorganisms related to maintaining soil health (Rahman, 2021). The comprehensive application of chemical fertilizer and organic fertilizer can affect many vital paddy ecosystem processes by regulating the soil microbial communities (Oladele et al., 2019). A recent study showed that long-term organic substitution increases rice yield by improving soil properties and regulating soil bacteria (Liu et al., 2021). Song et al. (2022) found that the organic substitution can reduce soil acidification, improve soil physicochemical properties and microbial communities, and enhance soil metabolism (Song et al., 2022). As an ideal source of organic fertilizer, *A. sinicus* can improve the nutrient cycle in soil and affect the fertilizer use efficiency of the soil (Yang W. et al., 2022). Ecological clusters are important ecological units, and microorganisms in the same ecological cluster have strong co-occurrence relationships (Heleno et al., 2012; Guidi et al., 2016). They provide an opportunity to investigate highly connected and identifiable taxa, and recent studies have concluded that ecological clusters and soil nutrient changes are closely related (Fan et al., 2021). Hence, to further understand the mechanism of paddy ecosystem changes, it is essential to study the response of soil microbial communities to *A. sinicus*.

Rice is one of the major food crops in the world (Kusano et al., 2015). According to the latest statistics from the Food and Agriculture Organization,^1^ rice ranks third in global food crop output. Thus, to meet the demand for food crops for a growing population, it is particularly important to maintain and ensure the health and stability of the rice field ecosystem. Chemical fertilizer application is one of the important ways to supply N nutrients to crops and enhance crop yield (Qian et al., 2016). However, the phenomenon of chemical fertilizer abuse is common, which endangers soil health and causes problems such as soil acidification, hardening, and fertilizer nutrient loss, which are not conducive to the sustainable development of agriculture (Liu et al., 2021; Song et al., 2022). The damage to soil health by unreasonable fertilization methods has caused widespread concern.

Organic fertilizer is rich in organic matter, which can effectively alleviate the soil problems caused by chemical fertilizer abuse and improve soil health (Jia et al., 2020; Yang W. et al., 2022). However, if only organic fertilizer is applied, its fertilizer effect will be exerted slowly, and it will be difficult to meet the expected crop yield increase in a short time (Garzón et al., 2011). Comprehensive application of organic and inorganic fertilizers may be an important measure to maintain the stability of the farmland ecosystem. Oladele’s et al. (2019) study showed that the combined application of carbon and organic fertilizer can improve the productivity of rice and the level of soil available nutrients. Liu et al. (2021) found that long-term organic substitution increases rice yield by improving soil properties and regulating soil bacteria. These studies show that organic substitution effectively alleviates the continuous deterioration of soil quality. Compared with the application of chemical fertilizer alone, organic substitution can significantly improve the utilization rate of N in fertilizer by crops (Cheng et al., 2021). However, excessive N fertilizer input may reduce the nutrient utilization rate of N input in the ecosystem, leading to N loss from soil to other environments and causing potential damage to the ecosystem (Zhao et al., 2019). *A. sinicus* is a well-established green manure for paddy soil in southern China. The moderate amount of *A. sinicus* returned to the soil can improve the protein content and overall quality of rice (Tong et al., 2022; Yang H. et al., 2022). A recent study showed that replacing urea-N with *A. sinicus* can mitigate N_2_O emissions in rice paddy (Yang W. et al., 2022). However, the influence of returning *A. sinicus* to paddy soil on the bacterial community remains poorly understood.

Here, 16S rRNA gene high-throughput sequencing technology was used to compare the bacterial community compositions (both taxonomic and functional diversity) between control and *A. sinicus* deposited samples. This study is based on a nine-year paddy field experiment performed by the Jiangxi Academy of Agricultural Sciences in 2008 with the objectives of determining (1) the bacterial community composition, structure, and assembly mechanism of paddy soil under different fertilization treatments; (2) the effects of fertilization on the correlations between microbes and environmental factors; and (3) the effects of different fertilization treatments on soil bacterial community function.

## Materials and methods

### Experimental design and sample collection

The experiment was set up in 2008 in Dengjiabu Rice Seed Farm, Yujiang County, Jiangxi Province, China (28°12′ E, 116°47′ N) with paddy soil formed by river alluvium. The annual temperature is 17.6°C, and the annual precipitation is 1788.8 mm. Four fertilization treatments with continuous rice cropping were compared in a completely randomized block design with three replicates (each plot was 20 m^2^): (1) CK: non-fertilization; (2) CF: 100% chemical fertilizer; (3) LA: 80% chemical fertilizer + moderate amount of *A. sinicus* (15,000 kg/hm^2^); and (4) HA: 80% chemical fertilizer + high amount of *A. sinicus* (37,500 kg/hm^2^). Generally, N fertilizer is urea, phosphate fertilizer is superphosphate, and potassium fertilizer is potassium chloride (N-P-K). The 100% chemical fertilizer comprised N (150 kg/hm^2^), P_2_O_5_ (75 kg/hm^2^), and K_2_O (120 kg/hm^2^). The equivalent N input of 15,000 kg/hm^2^ and 37,500 kg/hm^2^ of *A. sinicus* treated with LA and HA was 39.1 kg/hm^2^ and 97.8 kg/hm^2^, respectively.

After the rice harvest (April 2017), equal amounts of topsoil (0–20 cm) were collected from three experimental areas set in each treatment by multi-point mixed sampling. During soil collection, the soil moisture content was measured on the spot. The collected soil samples were temporarily stored in an ice box and immediately transported to the laboratory. Stones and weeds were removed from the sample as soon as possible, and the soil was passed through a 2-mm sieve. Each sample was then divided into two parts; one part was frozen at −80°C to be used later for DNA extraction, and the other was stored at 4°C for the determination of various physicochemical factors.

### Soil physicochemical analysis

Soil pH was measured by potentiometry (Coleman et al., 1951). The total organic matter (TOM) content was measured using the potassium dichromate volumetric method (Cai et al., 2011). Total nitrogen (TN) was determined by the Kjeldahl method (Craft et al., 1991). Alkali-hydrolyzable nitrogen (AHN) was determined by the diffusion method (Yang and Yang, 2020). Nitrate nitrogen (NN) was determined by ultraviolet (UV) spectrophotometry (Cawse, 1967). Ammonia nitrogen (AN) was determined by indophenol blue colorimetry (Kanda, 1995). Total phosphorus (TP) was determined by the molybdenum antimony colorimetric method (Wieczorek et al., 2022). Available phosphorus (AP) was determined by the sodium bicarbonate method (De Silva et al., 2015). Total potassium (TK) and available potassium (AK) were determined by flame spectrophotometry (Hald and Mason, 1958; Liu et al., 2021). To ensure the reliability of the experimental data, three independent measurements of these indicators were made for each sample.

### High-throughput sequencing and bioinformatics analysis

DNA was extracted from the samples using a MagPure Stool DNA LQ Kit (Magen, D6358-03, Guangzhou, China). To eliminate interference from external bacteria and impurities during the extraction, all experimental operations were performed according to the kit instructions at a UV-sterilized ultraclean bench (Bolyen et al., 2019). The V3–V4 regions of the bacterial 16S rRNA gene were amplified using the primers 341F (5′-CCTACGGGNGGCWGCAG-3′) and 805R (5′-GACTACHVGGGTATCTAATCC-3′). Amplification was performed with pre-denaturation for 3 min at 95°C, followed by 25 cycles of 30 s at 95°C, 30 s at 55°C, and 45 s at 72°C, and a final extension for 5 min at 72°C. PCR amplicons were detected by electrophoresis on 1.5% (w/v) agarose gels. The purified amplicons were sequenced on an Illumina MiSeq platform.

After sequencing, Quantitative Insights into Microbial Ecology 2 (QIIME2, v2020.8^2^) was used to process the offline data (original data) of different fertilization treatments under long-term unbalanced fertilization (Bolyen et al., 2019). Both ends of the original data were imported into QIIME2 as an input FASTQ file, and the semantic type of bacteria was specified as the single-end sequence. The quality of the raw sequence was evaluated, and low-quality cut-offs for forward and reverse reads were determined. QIIME2 was used to perform quality control and generate an ASV feature table. The quality control function in DADA2 was used for denoising and chimera detection and removal (Callahan et al., 2016). In addition, ASVs present in only one sample and a total relative abundance of less than five in all samples were removed. Subsequently, the ASV representative sequences were aligned with the SILVA database 138.^3^ To accurately assess the diversity of microbial communities, all samples were rarefied to the same depth based on the minimum sequence number (32,781). Subsequent analyzes performed in this study were based on the above normalized data.

### Statistical analysis

The ‘vegan’ package in R software was used to calculate the α-diversity (Chao1, abundance-based coverage estimator (ACE), Shannon, etc.) of the bacterial community. The ß-diversity (Bray–Curtis distance) of the bacterial community was calculated using the ‘betapart’ package. The ‘hmisc’ package was used to calculate Spearman correlation coefficients between the bacterial community and environmental factors or ecological niche width. The ‘NST’ package was used to quantify the assembly process of the bacterial community. The ‘rdacca’ package was used for hierarchical partitioning analysis. Origin software was used to draw box plots, scatter plots, and stacked histograms. The circular heatmap was visualized using the Tutools platform.^4^ The nonparametric factorial Kruskal–Wallis sum-rank test and linear discriminant analysis (LDA > 2.0) were used to identify biomarkers in the different treatments and were performed using the LEfSe tool in Galaxy/Hutlab.^5^ Partial least squares-discriminant analysis was performed using an online analysis platform.^6^ Based on the methods mentioned in previous studies (Langfelder and Horvath, 2008; Guidi et al., 2016; Chen et al., 2022), weighted gene correlation network analysis (WGCNA) was performed on the relative abundance bacterial community table. An online analysis platform^7^ was used to infer community assembly mechanisms by phylogenetic bin-based null model analysis (iCAMP). We assessed the normality of the distribution of indicators such as diversity using the Shapiro–Wilk index in IBM SPSS Statistics software and performed analysis of variance (ANOVA) and Duncan honestly significant difference test (*p* < 0.05) for data that conformed to a normal distribution and Kruskal–Wallis test (*p* < 0.05) for data that did not conform to a normal distribution.

## Results

### Soil physicochemical properties

Different fertilization treatments significantly affected the soil physicochemical properties such as TN, AHN, NN, AP, and TK (Table 1). The HA treatment significantly increased TN and AP in soil (*p <* 0.05). TN showed a significant increasing trend with the increase in *A. sinicus* returned to the soil (*p <* 0.05). Although the content of AHN, NN, and TK in soil also increased with the increase in *A. sinicus* returned to the soil, no significant difference was observed among treatments (*p >* 0.05). The AP content in soil of the LA treatment was the highest, which was significantly higher than the CK treatment (*p <* 0.05).

**Table 1:** Effects of different fertilization treatments on soil physicochemical properties.

	CK	CF	LA	HA
pH	4.797 ± 0.039	4.767 ± 0.074	4.743 ± 0.077	4.713 ± 0.037
SMC (%)	28.287 ± 0.901	28.077 ± 0.676	30.023 ± 2.095	30.233 ± 1.946
TOM (g/kg)	35.056 ± 2.18	36.603 ± 0.244	35.916 ± 1.199	35.419 ± 0.983
TN (g/kg)	1.879 ± 0.039d	1.93 ± 0.110c	2.01 ± 0.114b	2.086 ± 0.007a
AHN (mg/kg)	135.771 ± 4.042b	144.346 ± 16.169ab	155.779 ± 16.169ab	167.213 ± 0a
NN (mg/kg)	3.284 ± 0.673b	3.389 ± 0.805ab	3.645 ± 0.852ab	4.038 ± 0.321a
AN (mg/kg)	5.939 ± 0.815	5.647 ± 0.742	5.191 ± 0.31	5.051 ± 0.497
TP (g/kg)	0.402 ± 0.023	0.434 ± 0.051	0.502 ± 0.059	0.64 ± 0.149
AP (mg/kg)	1.643 ± 0.466b	4.091 ± 3.254ab	7.811 ± 4.995a	10.4 ± 2.171ab
TK (g/kg)	34.665 ± 2.092b	34.54 ± 2.166ab	36.125 ± 2.107ab	37.359 ± 3.335a
AK (mg/kg)	67.669 ± 13.057	69.092 ± 11.91	68.968 ± 11.996	59.961 ± 0.743

Different lowercase letters indicate differences among different treatments (ANOVA and Duncan’s test (*p* < 0.05) or Kruskal–Wallis test (*p* < 0.05)). pH, hydrogen ion concentration index; SMC, soil moisture content; TOM, total organic matter; TN, total nitrogen; AHN, alkali-hydrolyzable nitrogen; NN, nitrate nitrogen; AN, ammonia nitrogen; TP, total phosphorus; AP, available phosphorus; TK, total potassium; AK, available potassium.

### Bacterial α- and β-diversity

**Figure 1:**
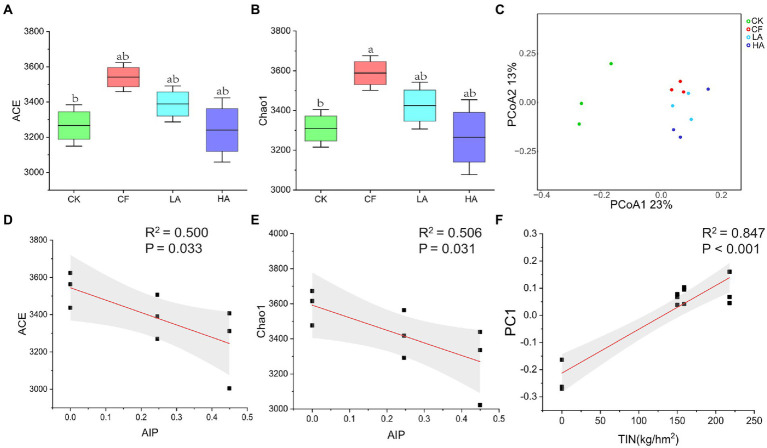
Soil bacterial community diversity and structure among different fertilization treatments. **(A)** Chao1 index of the bacterial community. **(B)** ACE index of the bacterial community. **(C)** ß-Diversity of the bacterial community. **(D)** Correlation analysis between AIP and Chao1. **(E)** Correlation analysis between AIP and ACE. **(F)** Correlation analysis between total inorganic N (kg/hm2) and ß-diversity. Different labeled letters indicate significant differences between different treatments according to one-way ANOVA with Duncan’s multiple range tests (*p* < 0.05).

The bacterial community in the various fertilization treatments showed different structural characteristics. Compared with the CK treatment, the CF treatment significantly increased α-diversity (ACE and Chao1) of the soil bacterial community (*p <* 0.05; Figures 1A, B). However, the Shannon index of the soil bacterial community did not change significantly (*p >* 0.05; Supplementary Table S1). The first two coordinates of the principal coordinate analysis (PC1 = 23% and PC2 = 13%) based on Bray–Curtis distance explained 36% of the variation in bacterial β-diversity. Principal coordinate analysis showed that ASVs were separated in overall treatment (Adonis test, *p* < 0.05; Figure 1C), implying that different fertilization treatments had significant effects on the bacterial structure. Regression analysis showed that with the increase in the input proportion of *A. sinicus* (the ratio of the N content of *A. sinicus* to total fertilizer N, AIP), the soil bacterial α-diversity (ACE and Chao1) significantly decreased (*p <* 0.05; Figures 1D, E). Differences in the bacterial community composition for all treatments were dominated by species replacement processes, which contributed 94.5%; whereas the contribution of richness difference to β-diversity was smaller (5.5%; Supplementary Table S2). With the increase in total inorganic N input, eigenvalues of the bacterial community structure (PC1) significantly increased (*p <* 0.001; Figure 1F).

### Composition of the soil bacterial community

In all samples, 40 phyla and 501 genera had certain taxonomic statuses. Among these, the average relative abundance of 12 phyla and 16 genera exceeded 1%, and were the dominant phyla and genera in this study. Acidobacteria (24.56%) and Proteobacteria (24.51%) were the dominant phyla in the soil bacterial community, followed by Chloroflexi (15.93%), Nitrospirota (8.16%), Bacteroides (5.79%), Myxococcota (4.24%), *Actinobacteria* (3.48%), Desulfobacterota (2.41%), Verrucomicrobia (2.07%), Gemmatimonadota (1.72%), Sva0485 (1.39%), and Firmicutes (1.18%). The relative abundance of Acidobacteria in the CK treatment was the highest. Compared with the other three treatments, the relative abundance of Proteobacteria and Firmicutes in HA treatment was significantly increased (*p <* 0.001; Supplementary Table S3).

### Correlations between environmental factors and bacterial community

**Figure 2:**
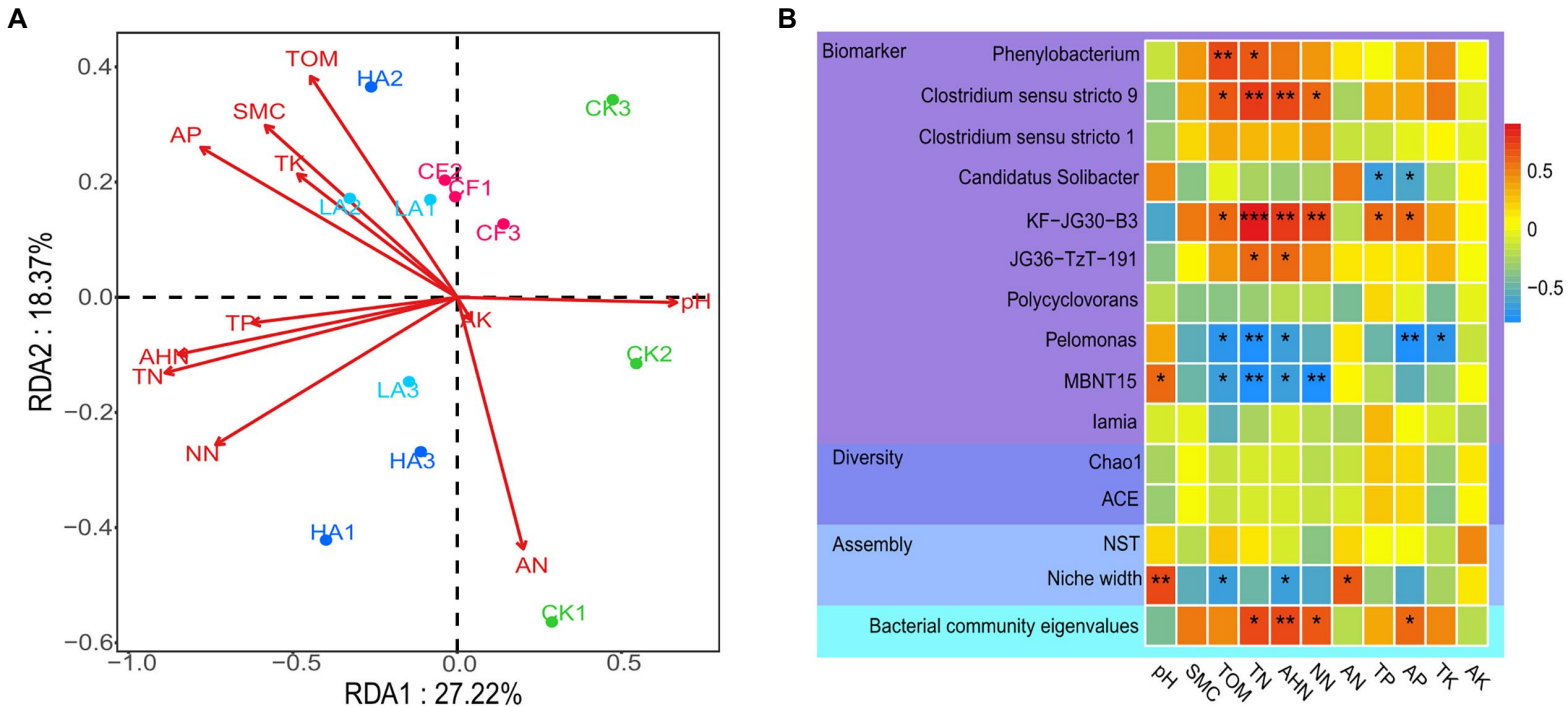
Relationships between the soil bacterial community and environmental factors. **(A)** Redundancy analysis between soil microorganisms and environmental factors. **(B)** Spearman correlation heatmaps of environmental factors and bacterial diversity, dominant genera, and bacterial community assembly. Red represents a positive correlation, and blue represents a negative correlation.

Redundancy analysis was performed to reveal the effects of environmental factors on all bacterial ASVs (Figure 2A), and hierarchical partitioning was used to obtain the explanation rate of each explanatory variable (Lai et al., 2022). The first two axes of the redundancy analysis accounted for 45.59% of the total variation in the bacterial community (PC1 = 27.22%, PC2 = 28.37%). The hierarchical partitioning analysis showed that among all environmental factors, AP (explanation rate = 12.55%) had the greatest influence on the bacterial community structure, followed by TN (explanation rate = 10.20%) and AHN (explanation rate = 10.20%; Supplementary Table S4). At the genus level, biomarkers that showed significant differences in the abundance between the different treatments were identified using the nonparametric factorial Kruskal–Wallis sum-rank test and linear discriminant analysis (LDA > 2.0; Liu et al., 2021). Spearman correlation analysis was performed to further characterize the relationships between biomarkers, bacterial diversity, bacterial community assembly, bacterial community eigenvalues, and environmental factors. Spearman correlations showed that TOM, TN, AHN, and NN had a significant positive correlation with *Clostridium sensu stricto 9*, *JG36-TzT-191*, and *KF-JG30-B3,* and a significant negative correlation with *MBNT15* (*p <* 0.05). TP and AP had a significant positive correlation with *KF-JG30-B3*, and a significant negative correlation with *Candidatus* Solibacter (*p <* 0.05). Furthermore, bacterial α-diversity (ACE and Chao1) and normalized stochasticity ratio (NST) did not show a significant relationship with each environmental factor. However, the niche width of the bacterial community was significantly positively correlated with pH and AN and negatively correlated with TOM and AHN. The content of AP, TN, AHN, and NN in soil was the most strongly correlated with bacterial community eigenvalues, which was consistent with the hierarchical partitioning analysis result (Figure 2B).

### Construction of ecological clusters

**Figure 3:**
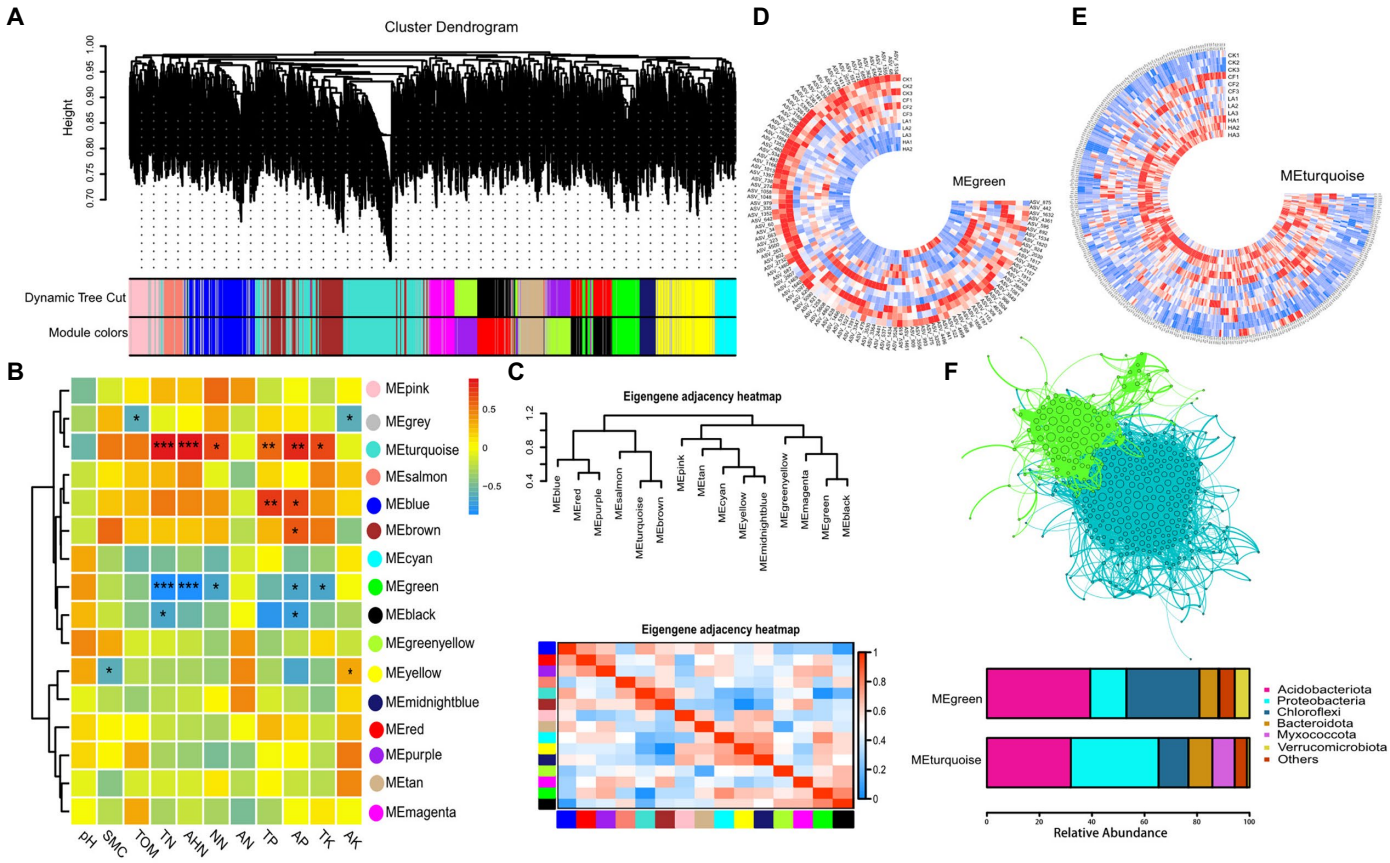
Ecological clusters generated by WGCNA. **(A)** WGCNA module plot. Dynamic tree cut represents initial clusters. Module colors represent final clusters. Each branch in the hierarchical tree or each vertical line in color bars represents one gene. Genes not attributed to any module are colored gray. **(B)** Pearson correlation heatmap between ecological clusters and physicochemical factors. **(C)** Pearson correlation heatmap between different ecological clusters. **(D)** Distribution of MEgreen in each treatment. Each color represents one module. **(E)** Distribution of MEturquoise in each treatment. **(F)** Network and species composition of MEgreen and MEturquoise.

Ecological clusters represent important ecological units that provide the opportunity to identify the highly connected and identifiable taxa (Heleno et al., 2012; Guidi et al., 2016; Fan et al., 2021). Here, WGCNA was applied to the relative abundance tables of prokaryotes, and the bacterial community was divided into 15 ecological clusters (Figure 3A). Pearson correlation analysis was performed to further investigate the relationships within different ecological clusters and between ecological clusters and physicochemical factors (Figures 3B, C). Two ecological clusters (MEturquoise and MEgreen) were strongly correlated with N levels in soil, with MEturquoise showing a significant positive correlation and MEgreen showing a significant negative correlation with soil N levels. Pearson correlation analysis between different ecological clusters showed that these two modules were significantly negatively correlated. The circular heatmap shows the distribution of the relative abundance of bacteria in MEturquoise and MEgreen across treatments. The relative abundance of bacteria in MEturquoise was lower in the *A. sinicus* return treatments (LA and HA), whereas the relative abundance of bacteria in MEturquoise was higher in the *A. sinicus* return treatments (Figures 3D, E). We then analyzed the species composition of the two ecological clusters and found that the bacteria were mainly from the phyla Proteobacteria, Acidobacteria, Myxococcota, Chloroflexi, Bacteroides, and Verrucomicrobia. Acidobacteria had the highest relative abundance in MEgreen, and Proteobacteria had the highest relative abundance in MEturquoise (Figure 3F).

### Assembly mechanisms of the bacterial community

**Figure 4:**
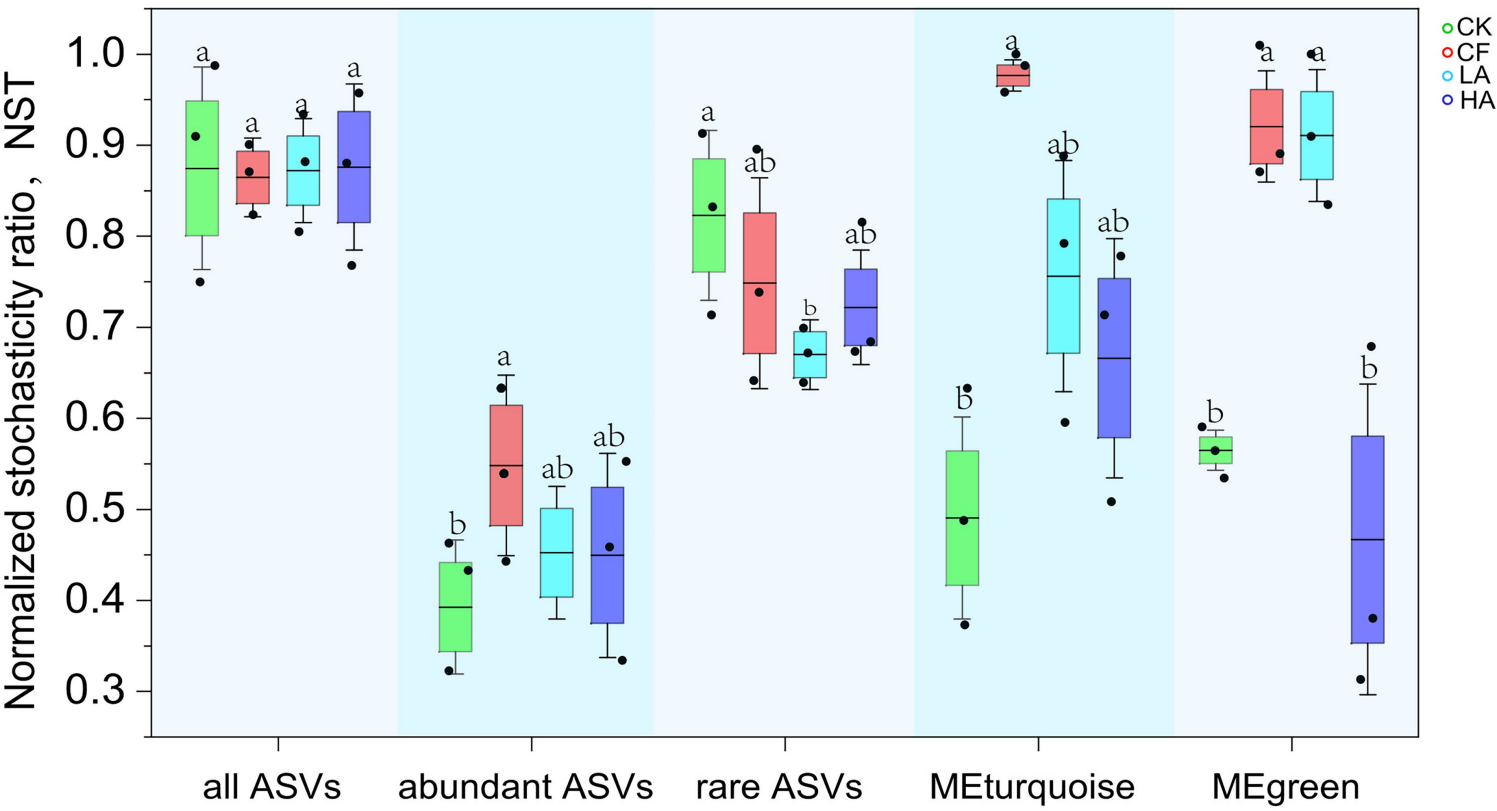
Normalized stochasticity ratio of all ASVs, abundant ASVs, rare ASVs, MEturquoise, and MEgreen in different treatments. Different labeled letters indicate significant differences between different treatments according to one-way ANOVA with Duncan’s multiple range tests (*p* < 0.05).

NST of all ASVs did not differ significantly in different treatments; thus, we further investigated NST of abundant ASVs (relative abundance ≥1% in all samples), rare ASVs (relative abundance <0.01% in all samples), MEturquoise, and MEgreen. The results indicated that NST of rare ASVs was higher than that of abundant ASVs, which meant that rare ASVs were more inclined to stochastic assembly. In contrast, deterministic processes contributed more to the assembly of abundant ASVs. NST of abundant and rare ASVs showed opposite variation trends, which explained why NST of all ASVs did not differ significantly. The LA and HA treatments decreased NST of abundant ASVs, rare ASVs, MEturquoise, and MEgreen. This, to some extent, implies that the stochastic assembly of rare and abundant ASVs in soil could be reduced by *A. sinicus* return to fields (Figure 4).

### Functional genes of ecological clusters

**Figure 5:**
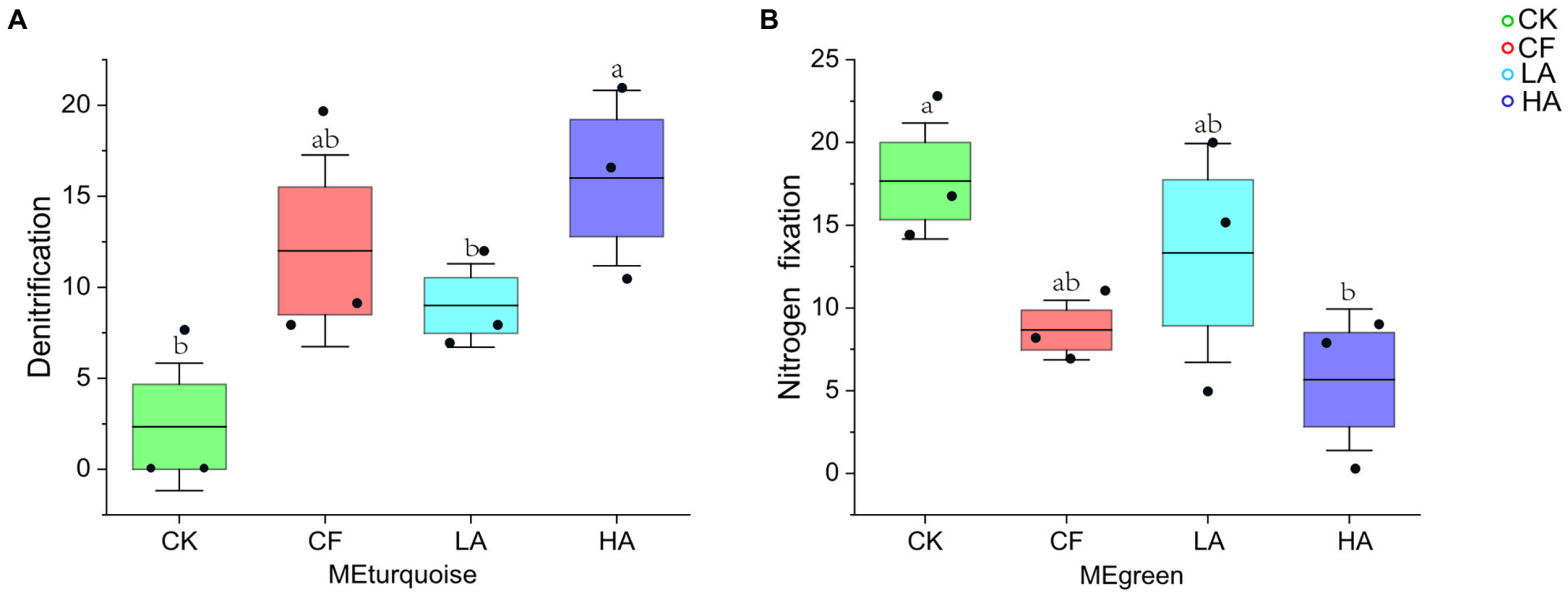
Functional abundance of ecological clusters. **(A)** Abundance of denitrification function in MEturquoise. **(B)** Abundance of N fixation function in MEgreen. Different labeled letters indicate significant differences between different treatments according to one-way ANOVA with Duncan’s multiple range tests (*p* < 0.05).

Functional annotation of prokaryotic taxa (FAPROTAX) is widely used for predicting functional profiles of biogeochemical cycling processes (especially C, H, N, P, and S cycling) in environmental samples (Xu et al., 2021; Sun et al., 2023). In this study, FAPROTAX was used to predict the function of key soil ecological clusters (MEturquoise and MEgreen). The relative abundance of denitrification function of MEturquoise significantly varied (Figure 5A), whereas the relative abundance of N fixation function did not vary significantly. The relative abundance of N fixation function of MEgreen significantly varied among treatments (Figure 5B). The results of the functional analysis showed that treatment without any fertilizer addition (CK) had the lowest relative abundance of denitrification function of MEturquoise and had the highest relative abundance of N fixation function of MEgreen (Figures 5A, B). Compared with CK, the relative abundance of N fixation function of key ecological clusters in the CF treatment was significantly reduced, and a moderate amount of *A. sinicus* returned to the soil instead of chemical fertilizer could effectively alleviate this process. The HA treatment caused a significant increase in the relative abundance of denitrification function of MEturquoise and a significant decrease in the relative abundance of N fixation function of MEgreen. A moderate amount of *A. sinicus* returned to the soil is more beneficial for key ecological clusters to increase the relative abundance of soil N retention function.

## Discussion

*Astragalus sinicus* is widely considered a well-established resource of organic fertilizer with high N content, and its partial replacement of chemical fertilizers can help improve the soil N pool (Yang W. et al., 2022), which was corroborated in this study. Partial replacement of chemical fertilizers by *A. sinicus* significantly increased soil *N* levels (TN, AHN, and AN) in addition to causing a significant increase in physicochemical indicators such as AP in soil. Increasing soil N and P levels is considered effective in improving crop yield and quality (Table 1; van Bruggen et al., 2015; Song et al., 2022). Although extensive *A. sinicus* returned to the soil plays an important role in improving soil nutrients, previous studies still poorly understood the changes in the bacterial community. Therefore, we focused on the effects of *A. sinicus* on the soil bacterial community, soil key ecological clusters, and functional abundance.

Microbial diversity is an essential feature of soil ecosystems, as microorganisms play a crucial role in nutrient cycling and maintenance of soil structure (Dai et al., 2018). The biodiversity of key ecological clusters was found to determine crop production in a 35-year fertilization experiment (Fan et al., 2021). Replacing chemical fertilizers with *A. sinicus* could lead to a decrease in α-diversity of the soil bacterial community (Figure 1) and an increase in the relative abundance of Proteobacteria (Supplementary Table S3). This conclusion was similarly confirmed by a previous meta-analysis, which concluded that N fertilizer application reduced bacterial diversity in global agroecosystems and favored the growth of Proteobacteria (Dai et al., 2018). Furthermore, the relative abundance of Acidobacteria was the highest in the CK treatment, which might be because of the oligotrophic state of the soil. These oligotrophic bacteria are thought to have the potential ability to break down nutrient-poor and refractory substrates, thereby promoting the growth of copiotrophic bacteria such as Alphaproteobacteria (Zhou et al., 2015). This also implies that *A. sinicus* returned to the soil may have shifted microbial life-history strategies, which transitioned from the K-strategy to the r-strategy (Shi et al., 2022). Therefore, the changes in the soil environment brought about by *A. sinicus* preferentially supported the growth of copiotrophic bacteria (e.g., some Proteobacteria) rather than oligotrophic bacteria (e.g., some Acidobacteria).

Soil physicochemical properties have long been recognized as important mediating factors in agricultural management (e.g., fertilization regimes) affecting soil microbial N response processes. Soil physicochemical conditions and N cycling processes of soil microorganisms are closely related. Compared with acidic and alkaline soils, neutral soils are of great positive significance for N-fixing microbial community structure stability and connectivity (Fan et al., 2018). Structural equation model analysis showed that soil total N and pH could indirectly regulate the primary N fixation rate and nitrification of soils by affecting soil microbial biomass C and increasing soil microbial biomass N, respectively (Lugtenberg, 2015). For denitrification, a high soil C/N content could promote the reduction of NO_3_^−^ to NH_4_^+^, while suppressing the N flow during denitrification to reduce soil N loss (Putz et al., 2018). The N content of soils is a good predictor of the soil denitrification function and volatilization of soil NH_3_ (Li Z. et al., 2022). Among the numerous soil physicochemical factors, some indicators (e.g., pH, N content) exhibited stronger influences on soil microbial N cycling process. Soil microbial communities are closely related to soil health, and the diversity and composition of microbial communities, in turn, can be largely influenced by environmental factors (Li M. et al., 2022). Therefore, it is important to study the relationship between microbial communities and environmental factors. Hierarchical partitioning can be used to solve the problem of covariance to obtain the contribution of individual explanatory variables to response variables (Lai et al., 2022). In hierarchical partitioning, N levels (TN, AHN, and NN) and AP in soil had the highest explanation rates for the bacterial community (Supplementary Table S4), implying that these indicators may be important environmental factors influencing the community structure. N and P are important environmental factors regulating the structure of soil microbial communities in rice fields, which have been widely verified in previous studies (Dong et al., 2014; Wang et al., 2021).

Ecological clusters are considered important ecological units, and microorganisms in the same ecological cluster have strong co-occurrence relationships, which provides an opportunity to investigate and explore highly related taxa within the total microbial community (Fan et al., 2021). Key ecological clusters are closely associated with ecosystems (de Menezes et al., 2015). To further investigate if ecological clusters of the bacterial community are more closely related to the soil environment, we applied the systems biology approach WGCNA to detect significant associations between the soil microbiome data and physicochemical factors. This method delineates clusters that are most associated with physicochemical factors (Langfelder and Horvath, 2008; Heleno et al., 2012; Guidi et al., 2016). Briefly, the WGCNA approach constructed a network and then clustered the network into modules (hereafter ecological clusters) that can be examined to find important ecological cluster–feature relationships (de Menezes et al., 2015). In this study, WGCNA was used to classify the bacterial community into 15 ecological clusters (Figure 3A). We found two ecological clusters (MEturquoise and MEgreen) that were most strongly correlated with soil N levels, with MEturquoise showing a significant positive correlation and MEgreen showing a significant negative correlation with soil N levels (Figure 3B). MEturquoise and MEgreen mainly contain Acidobacteria, Proteobacteria, and Chloroflexi (Figure 3F). We found a higher relative abundance of Proteobacteria in MEturquoise compared to MEgreen, which implies that *A. sinicus* returned to the soil may have shifted bacterial life-history strategies. The importance of these two ecological clusters among different treatments was further confirmed by partial least squares-discriminant analysis (Supplementary Table S5). In addition, the relative abundance of MEturquoise in soil gradually increased and that of MEgreen gradually decreased as the amount of *A. sinicus* returned to the soil increased (Figures 3D, E). This indicates that *A. sinicus* contributes to the growth of MEturquoise bacteria but inhibits the growth of MEgreen bacteria.

Investigating how microbial communities are assembled is crucial for understanding the response processes of microbial communities under organic substitution (Ning et al., 2020; Jiao et al., 2022). NST was used to quantify the extent of the contribution of stochastic assembly processes in the assembly of the bacterial community under different fertilizer application measures (Ning et al., 2019). Stochastic assembly is the dominant process driving the assembly of the entire soil bacterial community in our study. A higher contribution of stochastic assembly processes promotes the positive effect of microbial community diversity on multifunctionality (Li et al., 2021; Zhou et al., 2022). We further found that NST of rare ASVs was higher than that of abundant ASVs, implying that rare ASVs were more inclined to stochastic assembly processes. In contrast, the deterministic assembly process contributes more to the assembly of abundant ASVs. NSTs of abundant and rare ASVs showed opposite trends. The two parts of all ASVs (abundant and rare) reflected opposite trends, partially explaining why there was no significant difference in NST of total ASVs. Compared with the CF treatment, the *A. sinicus* return treatments (LA and HA) reduced NST of rare taxa, abundant taxa, MEturquoise, and MEgreen. This implies that the stochastic assembly process in the clusters could be reduced by *A. sinicus*. The relative contribution of deterministic and stochastic assembly processes may be related to the niche width of microbial communities. Microbial communities with larger niche widths may have greater metabolic plasticity and can utilize resources in the environment in a more balanced manner, less affecting the microbial communities by deterministic assembly processes (Chen et al., 2021). In addition, the contribution of processes such as homogeneous selection, heterogeneous selection, homogenizing dispersal, dispersal limitation, and ‘drift’ to the assembly process can be considered (Ning et al., 2020). Thus, we explored the factors that may contribute to differences in the stochastic assembly process between treatments by iCAMP analysis, but there was no significant variation in these indicators between treatments (Supplementary Table S6).

Microorganisms are important players influencing the biogeochemical N cycle (Guidi et al., 2016). Denitrification is one of the important biological processes for the removal of N in the biosphere. Denitrification by microorganisms dominates N loss in ecosystems and that N loss in soils due to denitrification cannot be ignored (Fang et al., 2015; Pan et al., 2022). Biological N fixation is a crucial ecological process and fixes up to 100 Tg N year^−1^ from the atmosphere on a global scale (Fan et al., 2019). Both denitrification function and N fixation function of microorganisms participate in determining the retention and removal of N in soil. We found that not all bacteria in ecological clusters significantly associated with the soil N level had N-related functions. Combined with the strong co-occurrence of bacteria in ecological clusters, we speculate whether there are “functional helpers” in the same ecological cluster that do not have the corresponding functions in the N cycle but can help N-cycling microorganisms to perform their functions and change the relative abundance of functional bacteria or influence their functions. The concept of microbial helpers has been mentioned in recent studies (Pan et al., 2022). However, the presence of functional helpers in the N cycle needs to be investigated and verified in more depth.

The application type of fertilizer can greatly affect the N response patterns of soil microorganisms (Lehnert et al., 2021). Compared with only N fertilization, N-P-K mixed fertilization can effectively alleviate the negative effect of N fertilization on soil bacterial diversity and is beneficial in promoting the mutualistic relationship between microorganisms and plants (Dai et al., 2018). In addition, straw returning is a common strategy to replace chemical fertilizers in agricultural systems and is essential for modulating the N response of the microbial community of agricultural systems. Straw return to fields can promote microbial contributions to soil N accumulation (Xia et al., 2021). Organic manure replacement mediated the contribution of soil microbial residues to the maintenance of soil N pools and enhanced soil microbial N fixation capacity, while competitively suppressing nitrification and reducing the risk of N loss (Ma et al., 2022). Thus, the type of fertilization largely influenced the N response processes of soil microbial communities and caused significant differences in the microbial communities in various ecosystems (Lugtenberg, 2015). Combined with these findings, the high N environment of the soil caused by returning *A. sinicus* might have caused the regulation of key ecological clusters for influencing the soil N content. Among all treatments, the CK treatment had the highest relative abundance of N fixation function of key ecological clusters, and when fertilizer was added to the soil, we found a significant increase in the relative abundance of the denitrification function and a significant decrease in the relative abundance of N fixation function of key ecological clusters, implying that the addition of fertilizer reduced the relative abundance of N fixation function of key ecological clusters. We continued to focus on the LA treatment and found that the application of a moderate amount of *A. sinicus* partially replacing chemical fertilizers could effectively alleviate the decrease in the relative abundance N fixation function of key ecological clusters. In contrast, if we continued to apply large amounts of *A. sinicus* to the field, the relative abundance in key soil ecological clusters (MEturquoise and MEgreen) for denitrification function was higher than the CF treatment, whereas that for N fixation function was lower than the CF treatment. This implies that a moderate amount of *A. sinicus* returned to the soil may be more beneficial for the key ecological clusters to maintain the soil N content, whereas a large amount of *A. sinicus* returned to the soil may promote N loss from the soil. In addition, these findings may explain, to some extent, why the TN content of LA and HA treatments significantly differed, whereas the NN content did not differ significantly. This may be because the HA treatment enriched the denitrification function, which promoted the conversion of NN to the other N forms. Previous studies using isotope labeling have observed that compared with chemical fertilizers, *A. sinicus* can significantly enhance the soil N pool to supply nutrients for the next crop round (Zhu et al., 2014; Yang W. et al., 2022). However, the fraction of N fertilizer inputs to the agroecosystem that exceeds the appropriate amount may have a significant impact on N emissions without contributing significantly to crop yield, which exacerbates global climate warming and ozone depletion (Rosas et al., 2015; Lehnert et al., 2021). A recent global meta-analysis showed that the increase in temperature changes the N cycle from microbial immobilization to nitrification and denitrification (Dai et al., 2020; Sun et al., 2022). This should make us realize that greenhouse gas emissions due to unreasonable farmland management will influence the N response of soil microorganisms, which may further promote the increase in NO_2_ emission factors and cause more serious N loss. The accumulative effect of this continuous cycling undoubtedly brings great pressure to the maintenance of the N fixation function of terrestrial ecosystems.

In brief, our study revealed the N response patterns of soil key ecological bacterial clusters in rice field ecosystems. In addition, it is worth noting that some recent studies have demonstrated that some beneficial soil fungi, such as arbuscular mycorrhizal fungi, can obtain NO_3_^−^, NH_4_^+^ and organic N from the surrounding soil and contribute to rice biomass (Panneerselvam et al., 2017; Wang et al., 2020; Das et al., 2022). These studies enlighten future research of plant-microbial interactions in rice field ecosystems. How to further optimize the ratio and input strategy of organic and inorganic fertilizers and find a good balance between fertilizer input benefit and ecological safety remains an extremely important research topic.

## Conclusion

This study focused on the response patterns of the soil bacterial community (especially key ecological clusters) to the *A. sinicus* returned to the soil. Soil N levels and AP were important environmental factors affecting the soil bacterial community in this study. We identified key ecological clusters (MEturquoise and MEgreen) by WGCNA. Partial replacement of fertilizer by *A. sinicus* had no effect on the assembly process of total ASVs (*p* > 0.05) but significantly enhanced the deterministic assembly process of abundant ASVs, rare ASVs, MEturquoise, and MEgreen (*p* < 0.05). The partial replacement of chemical fertilizers by moderate amounts of *A. sinicus* effectively mitigated the decrease in the relative abundance of N fixation function of key ecological clusters caused by chemical fertilizer application. However, an excessive amount of *A. sinicus* returned to the soil resulted in a significant increase in the relative abundance of denitrification function and a significant decrease in the relative abundance of N fixation function in key ecological clusters. In conclusion, our results imply that a moderate amount of *A. sinicus* returned to the soil can effectively mitigate the trend of reduced relative abundance of N fixation function of key ecological clusters caused by chemical fertilizer application.

## Data availability statement

The datasets presented in this study can be found in online repositories. The names of the repository/repositories and accession number(s) can be found at: https://www.ncbi.nlm.nih.gov/, PRJNA889571.

## Author contributions

JC, CX, and ML contributed to conception and design of the study. ML and YW performed the statistical analysis. WCS and WFS provided advice and guidance on the idea of bioinformatics analysis. XC and WQ participated in the sample collection. ML wrote the first draft of the manuscript. All authors contributed to the manuscript revision, read, and approved the submitted version.

## Funding

This study was funded by the National Key Research and Development Program of China (2021YFD1700203), the National Natural Science Foundation of China (31860592), the Innovation Fund of Jiangxi Academy of Agricultural Sciences, China (20182CBS002 and JXXTCXQN202008), and the National Industrial Technology System of Green Manure (CARS-22-Z-06).

## Conflict of interest

The authors declare that the research was conducted in the absence of any commercial or financial relationships that could be construed as a potential conflict of interest.

## Publisher’s note

All claims expressed in this article are solely those of the authors and do not necessarily represent those of their affiliated organizations, or those of the publisher, the editors and the reviewers. Any product that may be evaluated in this article, or claim that may be made by its manufacturer, is not guaranteed or endorsed by the publisher.

## Footnotes

^1^ https://www.fao.org/home/en

^2^ https://qiime2.org/

^3^ http://www.arb-silva.de/

^4^ http://www.cloudtutu.com/

^5^ http://huttenhower.sph.harvard.edu/galaxy/

^6^ http://www.biomicroclass.com/

^7^ http://ieg3.rccc.ou.edu:8080/

## Abbreviations

AHN: alkali-hydrolyzable nitrogen
AK: available potassium
AN: ammonia nitrogen
AP: available phosphorus
NN: nitrate nitrogen
SMC: soil moisture content
TK: total potassium
TN: total nitrogen
TOM: total organic matter
TP: total phosphorus
